# Presenteeism: The Invisible Leviathan of Organizational Psychology

**DOI:** 10.7759/cureus.48620

**Published:** 2023-11-10

**Authors:** K. Rakesh Chander, Madhan Jeyaraman, Naveen Jeyaraman, Sankalp Yadav

**Affiliations:** 1 Psychiatry, National Institute of Mental Health and Neurosciences, Bengaluru, IND; 2 Orthopaedics, ACS Medical College and Hospital, Dr. (MGR) Educational and Research Institute, Chennai, IND; 3 Medicine, Shri Madan Lal Khurana Chest Clinic, New Delhi, IND

**Keywords:** psychological benefits, organizational psychological medicine, workaholics, presenteeism, psychology

## Abstract

The burgeoning field of organizational psychological medicine identifies presenteeism, the practice of attending work while medically or psychologically unwell, as a complex factor influencing workplace health and overall organizational performance. This article examines presenteeism's many facets, focusing on how it affects the Indian labor force and how it increased during the COVID-19 epidemic, particularly in the field of healthcare. Utilizing data from the Ministry of Statistics and Programme Implementation and global surveys, the paper elucidates that an alarming percentage of the workforce abstains from utilizing entitled vacations, often leading to presenteeism. The article also probes the paradoxical aspect of presenteeism through the lens of "workaholism," arguing that while presenteeism may offer short-term psychological benefits such as boosting self-esteem or serving as a distraction from personal ailments, it is detrimental in the long term, impacting both individual health and organizational productivity. Recent surveys from the United Kingdom cited within the article indicate that presenteeism has tripled over the last decade, exacerbating health outcomes and compromising economic viability. Contributing factors are delineated, distinguishing between organizational imperatives such as financial penalties for absenteeism and individual motivations like job insecurity. The article ends by putting forward a multifaceted plan to mitigate the adverse consequences of presenteeism. The implementation of compassionate leadership, the adoption of flexible work practices, and the introduction of comprehensive employee well-being initiatives are among the key suggestions. Supervisors should also be trained in the identification and management of presenteeism. The article concludes by emphasizing the critical importance of strategic investment in human resources as a sustainable solution for curbing the detrimental impact of presenteeism on organizations.

## Editorial

Organizational psychological medicine is an upcoming branch of psychological medicine that deals with an organization's mental health and well-being, including its workforce. Workplace-related psychological well-being is influenced not only by internal incidents within the organization but also by external factors [1]. Certain factors from the former and the latter can demoralize human resources and thereby negatively impact the overall performance and efficiency of the workplace. Organization-related factors [2] are (i) unclear vision and objectives; (ii) poor communication and management practices, including leadership; (iii) inadequate support among employees; (iv) inflexible working hours; (v) limited participation of employees in decision-making; and (vi) inadequate health and safety policies. Factors beyond the organization [2] are (i) societal pressure and needs of the employees; (ii) their personality and competency in handling work-life-related stressful events; and (iii) employees having an existing mental health condition like depression and anxiety. Either group of the factors mentioned above shall impact the organization's outcomes, thereby leading to a loss in credibility and finance. The interplay of internal and external factors can precipitate various psychopathological phenomena within the workplace environment [1]. These phenomena may include embitterment, presenteeism, burnout, stress, and corporate responsibilities, among others. This article focuses on discussing the impact of presenteeism within the workplace ecosystem.

Presenteeism and labour

The International Labour Organization (ILO) defines 'work' as productive labor carried out in conditions of freedom, equity, security, and human dignity. An employee is defined as a person who has either worked for pay or profit for at least one hour during a specific week or who has a job but is currently absent due to reasons such as sickness, holiday, or maternity leave, among others. As per the Ministry of Statistics and Programme Implementation data for 2022, around 47% of the Indian population is involved in the country's labor force [3]. A recent global survey reported that India is one of the most vacation-deprived countries in the world [4]. Around 55% of Indians take fewer vacations than they are entitled to. This includes both annual and short vacations, with the latter observing only 2% of those availing of the monthly leave benefits. The survey found that 37% of them check emails and voicemails while on leave, and 67% cancel or postpone holiday plans, citing work-related reasons and fear of cumulating work post-vacation [5]. Other reasons reported were feeling guilty about taking leaves, fear of criticism from the management or their bosses, saving leaves for encashments, and future major events, including health concerns. However, it was found that only 17% of the Indians used their vacations for the latter [4]. In organizational psychology, 'presenteeism' or 'sickness presenteeism' means continuing to work while unwell [6]. Studies from around the world have documented presenteeism as a common incidence across cultures [7]. It would not be an exaggeration to state that the recent COVID-19 pandemic era witnessed a significant increase in presenteeism, especially in the health sector [8]. This occurrence is despite the recession in the market and the sacking of employees during the pandemic.

The workaholics’ argument

Though presenteeism is expected to bring in more productivity at work, research in the field has highlighted otherwise time and again. Being present despite not being able to perform optimally can still be beneficial. It has been argued to boost self-esteem and confidence as a sense of distraction from the ailment, a feeling of being socially engaged and supported during the suffering [8,9]. This holds true for illnesses that are chronic and disabling, like cancer, diabetes, etc. It also serves as a distractive strategy for employees experiencing psychological distress secondary to couple, family, and other related conflicts. Even in cases of mild-to-moderate severity of clinically diagnosed major depressive disorders, attending work is prescribed as a part of behavioral therapy called 'behavioral activation.' This suggests that the workplace can turn out to be a therapeutic environment during such scenarios. In addition, employees who are competitive and career-oriented tend to minimize their health hazards and continue to work. Some of them are self-motivated due to their personality traits, and the rest seem to be governed by the organization's 'Reward Management' policy [6]. Moreover, certain health conditions can result from working overtime, extra shifts, spending time at home for official work, and working on vacations. This, in turn, can not only lead to the risk of acquiring physical illness but also strain personal relationships [6]. The term 'workaholism' is defined as a strong obsession or need to work in an individual to the extent that it creates disturbances in his or her health and happiness, interpersonal relations, and social functioning. Thus, it is understood that workaholics (individuals who show signs of workaholism) do indulge in sickness presenteeism for a sense of self-appraisal and anticipation of reward [6].

The invisible leviathan

Having said that, the quality of work and productivity of these workers is questionable when they are present during sickness. Recent surveys conducted among professionals and business leaders during the COVID-19 pandemic in the United Kingdom (UK) reported that more than three-fourths of the respondents experienced delayed recovery, posing a risk of contagious transmission to their colleagues [9]. Even the strategic move of 'working from home' during the lockdown diffused the delineation between office hours and personal time, making the case worse by denting their mental health. This has resulted in considerable losses and a decline in the growth of both organizations and the individuals involved. Generally, presenteeism leads to an average loss of more than 4000 euros and two weeks per year per employee in the UK [9]. Working exhaustively while sick eventually leads to worsening symptoms and poor recovery, thereby lengthening the duration of suffering and causing burnout. Thus, presenteeism sometimes leads to sickness absenteeism, which could be longer than the intended leave period in the first place. The employee may inadvertently engage in a compensatory working spree, demonstrating compensatory workaholism that might result in presenteeism [9]. This statement suggests that presenteeism could very well cause a vicious cycle, causing repeated economic constraints.

Contributing factors

As mentioned earlier, a lot of factors contribute to this phenomenon among employees at various levels. Fundamentally, an individual needs to earn money to sustain a healthy, economically secure, and dignified lifestyle. With the world moving towards modernization and digital technology, there is a surge of novel start-ups, especially in the developing part of the world. This has led to the emergence of critical recruitment processes so that agile and competitive candidates can be hired. Hence, the need for an ever-achieving attitude from either party (the employee or the employer) has led to avenues for such phenomena (presenteeism, workaholism, etc.). In addition, certain sectors do warrant an increased workload and working hours with fewer vacations due to poor demand-supply. The following are examples under each group of factors that contribute to presenteeism [9], as mentioned in Table 1.

**Table 1:** Contributing factors for presenteeism. Source: [9]

Organization-related factors	Individual-related factors
Financial penalties for absenteeism	Primary breadwinner of the family
Stigmatizing sick leaves to pressure attendance	Financial constraints or worries
Poor supply (inadequate staffing), fearing a recession	Job insecurity and fear of being micro-scrutinized
Multiple roles and responsibilities	The feeling of guilt, indispensableness, or excessive loyalty
Unhealthy working relationships (including harassment based on sick leaves)	Conscientious nature (meeting personal standards)
Uncompassionate leadership	Business owner or chief executive of the firm

Ways to curtail the demon

To achieve and sustain the desired outcome of an organization, there has to be an ethical and implicit mechanism to keep phenomena such as presenteeism in check. Though various committees address the employees' grievances, such factors tend to be more intrinsic to make note of until the management is sensitive and proactive. Orienting supervisors and managers and training them to deal with such issues (including identification and periodic risk assessment of employees) shall prove vital in prevention at the first level. Compassionate leadership that considers flexible working hours and emphasizes quality rather than quantity of work time would be pivotal in improving workplace culture. Employee well-being programs with recreation and team-building activities will also add to the healthy work ecosystem. Transparency in the communication of the recruitment process by both parties in their roles and responsibilities, regular amendments of sickness and work policies, improving access, and minimizing barriers to health care (including psychological health) will go a long way in changing the trends of presenteeism and other such phenomena. Presenteeism, as a phenomenon, can be both beneficial and detrimental to an individual or an organization. Recognizing its emergence as a 'demon' that can lead to economic and resource depletion is crucial. Utilizing resources optimally and thereby providing a healthy working environment is the key factor in deciding the sustainability of a corporation. Thus, investing wisely in employees is the need of the hour for a Human Resource Management department, which has to be done pragmatically to suit the current trends.
